# Breaching septal veins while attempting left bundle branch area pacing. If necessary, septography contrast should be injected in a stepwise approach

**DOI:** 10.1002/joa3.12942

**Published:** 2023-10-13

**Authors:** Andrés Di Leoni Ferrari, David Santacruz Pacheco, Fabiano Segat, Diego Chemello

**Affiliations:** ^1^ Hospital São Lucas, Pontifícia Universidade Católica do Rio grande do Sul (PUCRS) Porto Alegre Brazil; ^2^ Fundación Clínica Shaio Bogotá Colombia; ^3^ Cardiodiagnóstico, Hospital de Caridade Astrogildo de Azevedo Santa Maria Brazil; ^4^ Instituto do Coração de Santa Maria (ICOR) Santa Maria Brazil; ^5^ Postgraduate Program in Gerontology Universidade Federal de Santa Maria (UFSM) Santa Maria Brazil

**Keywords:** interventricular septum, left bundle branch area pacing, pacemaker, septal veins fistula

## Abstract

Permanent transseptal left bundle branch area pacing (LBBAP) is a promising technique developed to avoid the detrimental effects of pacing‐induced dyssynchrony with right ventricular (RV) pacing, by offering more physiologic activation of the heart. Lesions to tributary veins of the coronary sinus have been increasingly reported, mostly associated with venous fistula or venous septal system infringement. Despite being mostly benign, venous complications may be related to the maneuver of contrast injection through the sheath and failure to follow simple but essential steps.

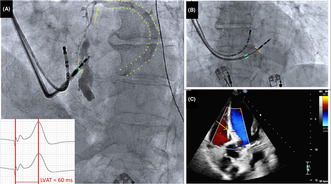

Permanent transseptal left bundle branch area pacing (LBBAP) is a promising technique developed to avoid the detrimental effects of pacing‐induced dyssynchrony.^1^ It was first described by Huang et al. to overcome the limitations of His‐bundle pacing.^2^ It requires the pacing lead to be screwed deep into the interventricular septum (IVS) aiming to achieve the layer below the left ventricular (LV) subendocardial region to capture the left bundle or its branches.^2^ Registries have shown high implant success. Complications can reach up to 16.4%, and some are related to the IVS, including septal myocardial injury, perforation into the LV cavity, atrioventricular block (AVB), lesions to the coronary artery branches, and septal hematoma.^3^ Lesions to tributary veins of the coronary sinus (venous fistulas or venous septal system infringement) have also been reported. These complications are usually related to the maneuver of contrast injection through the sheath and failure to follow simple but essential steps.^4^

We present two cases with venous system infringement after LBBAP. Both were made during contrast injection through the sheath after screwing the pacing lead deep into IVS. We discuss important steps that could prevent such issues. We also challenge the practice of contrast injection during septography to confirm lead position in cases where electrocardiographic parameters reveal good LBBAP.

In the first case, an 89‐year‐old woman presented for consultation due to recent‐onset fatigue. Her heart rate was 32 beats/min with blood pressure of 142/86 mmHg. The electrocardiogram revealed type 2 second‐degree AVB. There were no signs of coronary artery disease on angiogram. Her echocardiogram showed left ventricular ejection fraction (LVEF) of 63%. The electrophysiologic study confirmed second‐degree infra‐Hisian AVB. Left bundle branch area pacing was performed with a 5.6‐F stylet‐driven pacing lead with extendable helix (Solia S60, Biotronik, Berlin, Germany) delivered through a preshaped sheath (Selectra3D, Biotronik). Nonselective LBBAP was obtained (threshold 0.7 V at 0.4 ms) and stimulation to peak left ventricular activation time (LVAT) of 62 ms (Figure 1A). Unipolar pacing impedance was 689 ohms. Ten milliliters of contrast was injected through the sheath to confirm deep septal implantation. During injection, flow of contrast material was observed from a septal venous branch into the coronary sinus (CS). A small area of septal staining was also noted. (Figure 1A; Video S1). After withdrawing the sheath 1 cm away from the IVS, another injection was performed with 4 mL of contrast, which did not reveal transit to septal branches (Figure 1B; Video S2). A transthoracic echocardiogram confirmed septal lead position and no fistula formation (Figure 1C). There was a transient increase in high‐sensitivity Troponin (556 ng/L, normal values <39.5 ng/L). The chest X‐ray confirmed the appropriate lead position (Figure 2A,B), and the ECG revealed nonselective LBBAP (Figure 2C). At 3‐month follow‐up, the patient remained stable.

**FIGURE 1:**
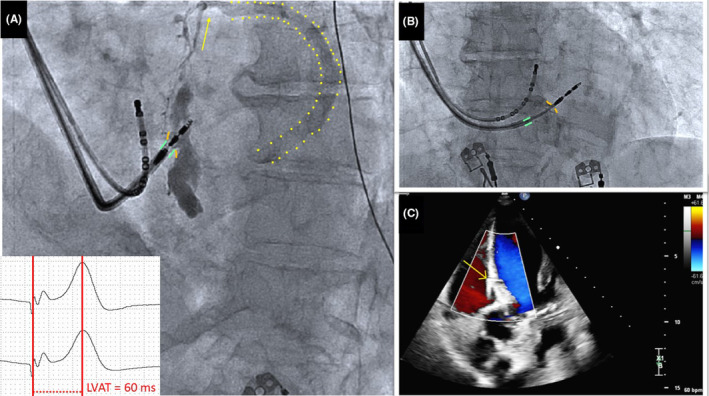
Case 1. (A) Angiography showing the close proximity between the sheath and the interventricular septum (green and orange marks), as well as the coronary sinus branch (yellow arrow) and coronary sinus (yellow dots) during contrast injection. Left anterior oblique projection. The stimulation to peak left ventricular activation time (LVAT) measurement was 62 milliseconds. (B) After withdrawing the sheath 1 cm, another injection was performed with 4 mL of contrast, which did not reveal residual contrast material or transit to septal branches withdrawn. The green marks reveal the tip of the sheath, while the orange marks show the interventricular septum. Left anterior oblique projection. (C) The echocardiogram postprocedure revealed lead position into the interventricular septum (yellow arrow) and no signs of fistula.

**FIGURE 2:**
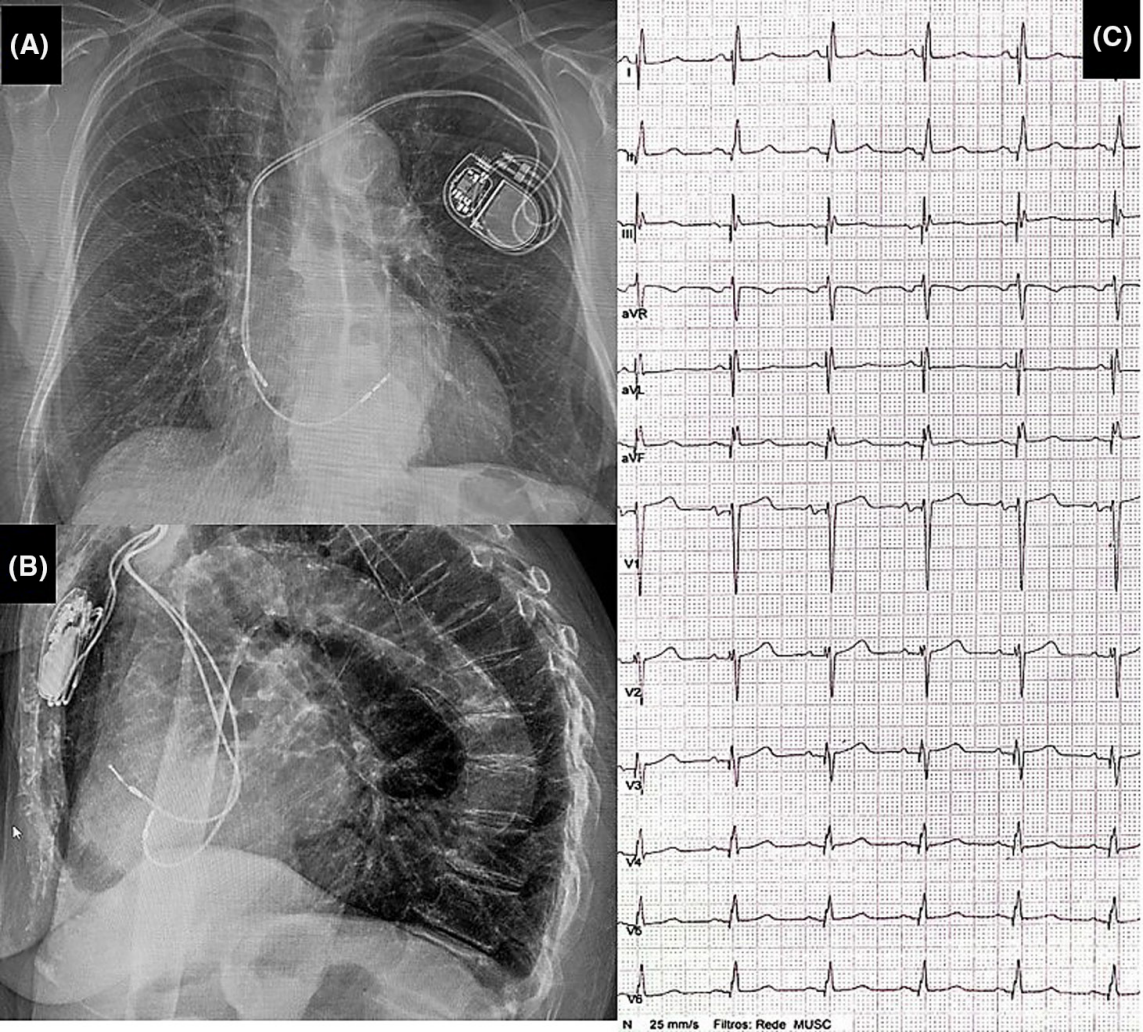
Case 1. (A and B) Chest X‐ray showing lead position in anteroposterior (A) and lateral (B) view. (C) Final 12‐lead ECG after left bundle branch area pacing implantation.

In the second case, a 72‐year‐old man with Chagas cardiomyopathy (LVEF 17%) was admitted due to recurrent implantable‐cardioverter defibrillator (ICD) shocks. He had class III NYHA functional class despite optimal medical therapy. He also received a dual‐chamber ICD implantation years ago. Device interrogation showed multiple episodes of ventricular tachycardia (VT) treated with appropriate shocks.

There was a ventricular pacing burden >80% because of baseline intermittent 2:1 AVB. Baseline ECG registered atrial paced activity, right bundle branch block, native QRS width of 150 ms, and first‐degree AVB (Figure 3A). Given all these findings, ablation of the LV arrhythmic substrate was performed. Cardiac resynchronization therapy (CRT) was performed to improve outcomes. After an unsuccessful attempt at CS cannulation, LBBAP was performed by implantation of a new ventricular lead, which was connected to the sense/pace terminal of the ICD. This decision was in accordance with recent guidelines.^5^ We used a steerable sheath (Agilis HisPro, Abbott, Chicago, USA) to implant a 5.8‐F stylet‐driven pacing lead (2088 Tendril, Abbott, Chicago, USA). During the attempt at deep septal penetration, a 10 mL contrast injection through the sheath revealed the lead tip and helix entering a branch of the coronary sinus (Figure 3C; Video S3). After retracting the helix and the lead, a more apical placement of the deep transseptal lead was achieved. Another 5 mL of contrast was injected while retracting the sheath, without signs of septal vein infringement. Nonselective LBBAP was obtained (threshold 1.0 V at 1.0 ms, LVAT 76 ms). Unipolar pacing impedance was 702 Ω. At follow‐up, there was an improvement in his NYHA functional class. Twelve‐lead ECG revealed a nonselective LBBAP (Figure 3B).

**FIGURE 3:**
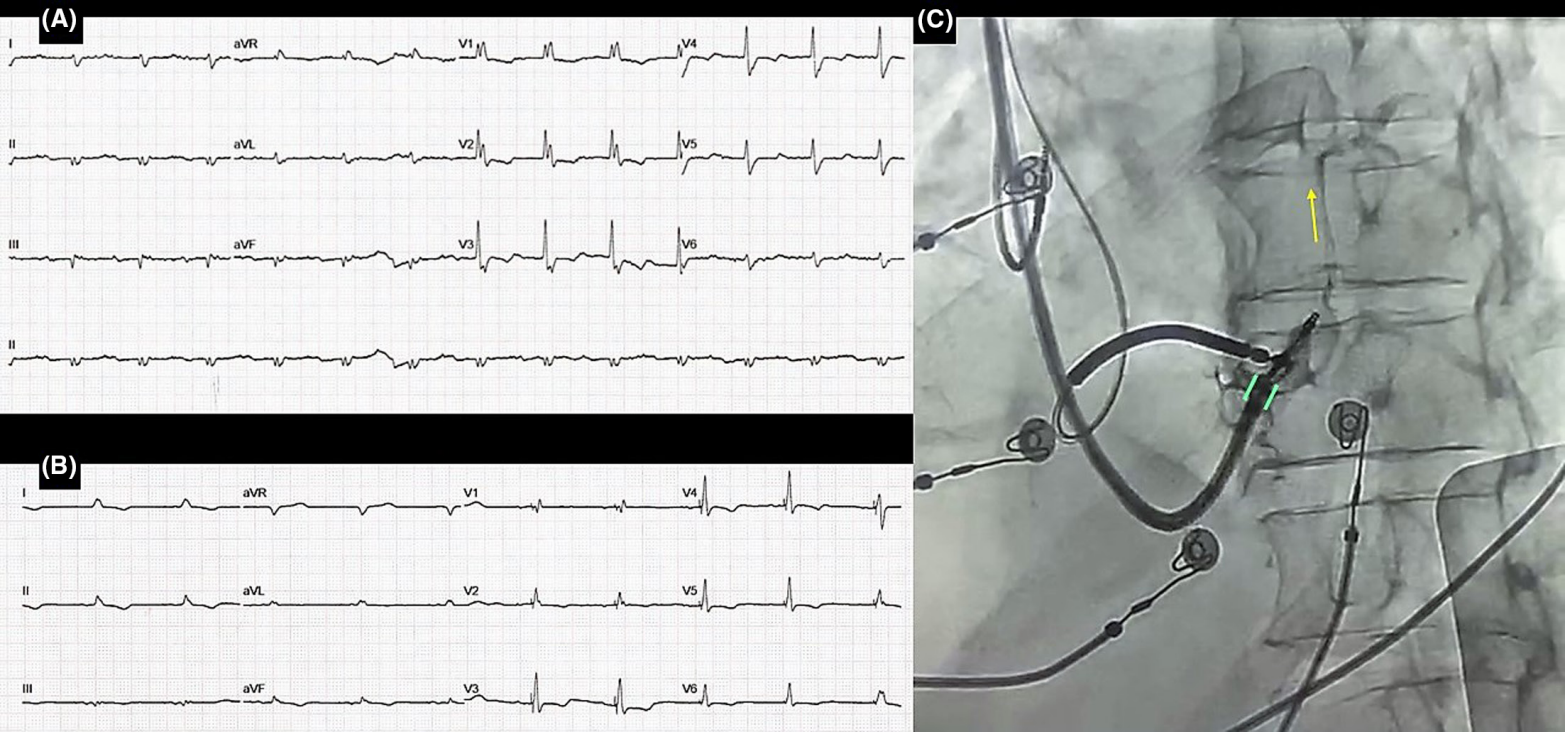
Case 2. (A) The baseline 12‐lad ECG with first‐degree AV block and right bundle branch block. (B) Final 12‐lead ECG after left bundle branch area pacing implantation. (C) Angiography in the left anterior oblique view showing the close proximity between the sheath and the interventricular septum (green marks), as well as the coronary sinus branch (yellow arrow) during contrast injection.

Septal coronary vein infringement during LBBAP is considered rare, although probably underdiagnosed. With rapid adoption of LBBAP, case reports of cardiac venous infringement have been increasing. Batul et al. reported two cases of venous drainage through cardiac venous system during LBBAP, using both stylet‐driven and lumenless fixed helix leads.^4^ The authors also reviewed 340 cases and observed this phenomenon in 9 (2.6%) cases.^4^ In the multicenter MELOS Study, so far the largest registry in the field, only seven cases of coronary vein fistulas out of 2533 LBBAP procedures (0.28%) were described.^3^

The common aspect of the reports of coronary venous penetration is the absence of serious complications or pacing parameters. This is probably related to the small size of the fistulas. Despite some operators opting to reposition the lead, one should question whether this step is necessary. Some authors have hypothesized that small‐diameter lumenless leads might reduce the risk, which was not confirmed. It seems that lead penetration, as well as the inappropriate contrast injection technique may precipitate venous infringement.^4^

Operators performing LBBAP should consider some steps before contrast injection to confirm the lead position in the interventricular septum. We avoid venous septum injury by (1) pulling the sheath slightly backward from the IVS and confirm sheath position in fluoroscopy; (2) using a small volume of nondiluted contrast (2 mL) injected gently and slowly. Following this stepwise approach, we may reduce the chance of high contrast pressure toward the IVS. Operators should also question whether is necessary to perform septography in cases with good electrocardiographic parameters signaling LBBAP. However, if they consider that contrast injection is essential, they should perform it in a stepwise approach.

## FUNDING INFORMATION

There was no funding source.

## CONFLICT OF INTEREST STATEMENT

Authors declare no conflict of interests for this article. Patients provided full written consent.

## Supporting information


Video S1.

Video S2.

Video S3.
